# Assessment of malaria transmission intensity and insecticide resistance mechanisms in three rural areas of the Moyen Ogooué Province of Gabon

**DOI:** 10.1186/s13071-022-05320-9

**Published:** 2022-06-20

**Authors:** Stravensky Térence Boussougou-Sambe, Tamirat Gebru Woldearegai, Ange Gatien Doumba-Ndalembouly, Barclaye Ngossanga, Romuald Beh Mba, Jean Ronald Edoa, Jeannot Fréjus Zinsou, Yabo Josiane Honkpehedji, Ulysse Ateba Ngoa, Jean Claude Dejon-Agobé, Steffen Borrmann, Peter G. Kremsner, Benjamin Mordmüller, Ayôla A. Adegnika

**Affiliations:** 1 Centre de Recherches Médicales de Lambaréné, Lambaréné, Gabon; 2 Institut für Tropenmedizin, Eberhard Karls Universität, Tübingen, Germany; 3 Department of Parasitology, Leiden University Medical Center, Leiden, the Netherlands; 4 Center of Tropical Medicine and Travel Medicine, Department of Infectious Diseases, Division of Internal Medicine, Academic Medical Center (AMC), University of Amsterdam, Amsterdam, the Netherlands; 5 German Center for Infection Research (DZIF), partner site Tübingen, Tübingen, Germany; 6 Department of Medical Microbiology, Radboud University Medical Center (UMC), 6524 GA Nijmegen, The Netherlands; 7 Fondation pour la Recherche Scientifique (FORS), Cotonou, Benin

**Keywords:** *Anopheles gambiae* complex, *Plasmodium* species, Entomological inoculation rate, Moyen Ogooué Province, Gabon

## Abstract

**Background:**

Vector control is considered to be the most successful component of malaria prevention programs and a major contributor to the reduction of malaria incidence over the last two decades. However, the success of this strategy is threatened by the development of resistance to insecticides and behavioural adaptations of vectors. The aim of this study was to monitor malaria transmission and the distribution of insecticide resistance genes in *Anopheles* populations from three rural areas of the Moyen Ogooué Province of Gabon.

**Methods:**

*Anopheles* spp. were collected using human landing catches in Bindo, Nombakélé and Zilé, three villages located in the surroundings of Lambaréné, during both the rainy and dry seasons. Mosquitoes were identified morphologically, and DNA was extracted from heads and thoraces. Members of the *Anopheles gambiae* complex were identified by molecular methods using the PCR SINE200 protocol and by sequencing of the internal transcribed spacer 2 region. Taqman assays were used to determine *Plasmodium* infection and the presence of resistance alleles.

**Results:**

*Anopheles gambiae* sensu lato (97.7%), *An. moucheti* (1.7%) and *An. coustani* (0.6%) were the three groups of species collected. *Anopheles gambiae* sensu stricto (98.5%) and *An. coluzzii* (1.5%) were the only species of the *An. gambiae* complex present in the collection. Of the 1235 *Anopheles* collected, 1193 were collected during the rainy season; these exhibited an exophagic behaviour, and consistently more mosquitoes were collected outdoor than indoor in the three study areas. Of the 1166 *Anopheles* screened, 26 (2.2%) were infected with *Plasmodium* species, specifically *Plasmodium falciparum* (66.7%), *P. malariae* (15.4%), *P. ovale curtisi* (11.5%) and *P. ovale wallikeri* (3.8%)*.* Malaria transmission intensity was high in Zilé, with an average annual entomological inoculation rate (aEIR) of 243 infective bites per year, while aEIRs in Bindo and Nombakélé were 80.2 and 17 infective bites per year, respectively. Both the *L1014F* and *L1014S* mutations were present at frequencies > 95% but no *Ace1G119S* mutation was found.

**Conclusion:**

Our results demonstrate that malaria transmission intensity is heterogeneous in these three rural areas of Moyen Ogooué Province, with areas of high transmission, such as Zilé. The exophagic behaviour of the mosquitoes as well as the high frequency of resistance mutations are serious challenges that need to be addressed by the deployment of control measures adapted to the local setting.

**Graphical Abstract:**

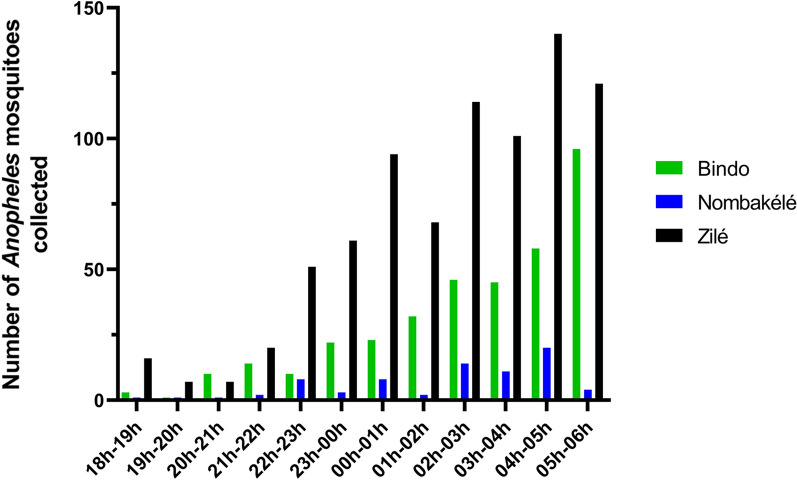

**Supplementary Information:**

The online version contains supplementary material available at 10.1186/s13071-022-05320-9.

## Background

Malaria remains a major public health issue in many malaria-endemic countries. Despite all the efforts put in place to control malaria, the disease still took an estimated 409,000 lives in 2019, mostly children under 5 years of age in sub-Saharan Africa [1]. Between 2000 and 2015, the incidence of malaria due to *Plasmodium falciparum* decreased by 40%, which a significant proportion of this decrease attributable to malaria vector control interventions, such as insecticide-treated nets (ITNs) and indoor residual spraying (IRS) [2]. However, there is evidence that members of the *Anopheles gambiae* complex and the *Anopheles funestus* group, the main malaria vectors in Africa [3], have developed physiological resistance to the insecticides used in malaria control programmes. Pyrethroids were until recently the sole class of insecticides used to impregnate bednets, and this is now a main factor driving the emergence and spread of insecticide resistance [4]. Furthermore, while the widespread use of ITNs has had a large effect in reducing endophilic/anthropophilic vectors, some evidence indicates that it may have led to changes in vector behaviour and mosquito population composition. Consequently, some malaria vectors have changed their biting activity from indoors to outdoors as well as their biting time, which reduces the effect of the two main interventions [5].

There is a need for data on local vector species prior to or following the introduction of vector control measures. In Gabon, ITNs are the main tools used in vector control, however, net ownership in 2019 was estimated to be below 20% [1]. Moreover, data on malaria transmission are lacking as only few entomological assessments have been carried out over the years [6–9] and distribution of insecticide resistance has received little attention [8–11]. Most of the studies carried out to date focussed mainly on a few parts of the country, with members of the *An. gambiae* complex identified as the main malaria vectors in Lambaréné, Libreville and Port-Gentil [7–9], and *An. funestus* found to be the primary and secondary vector in Akou and Benguia, respectively [6, 12]. Other mosquito species, such as *Anopheles nili*, *An. moucheti* and *An. hancocki*, have been reported to be secondary vectors [6, 7, 12].

Sylla et al. [7] reported that *An. gambiae* and *An. moucheti* are the main vector species in Moyen Ogooué Province, situated in the midwestern part of Gabon. However, no data are currently available on the distribution of insecticide resistance genes in malaria vectors. The aim of the present study was to assess species composition of malaria vectors, sporozoite rate, *Plasmodium* species composition, entomological inoculation rate (EIR) and presence of insecticide resistance genes in localities where intense clinical malaria research activities have been carried out for > 25 years [13].

## Methods

### Study sites

**Fig. 1:**
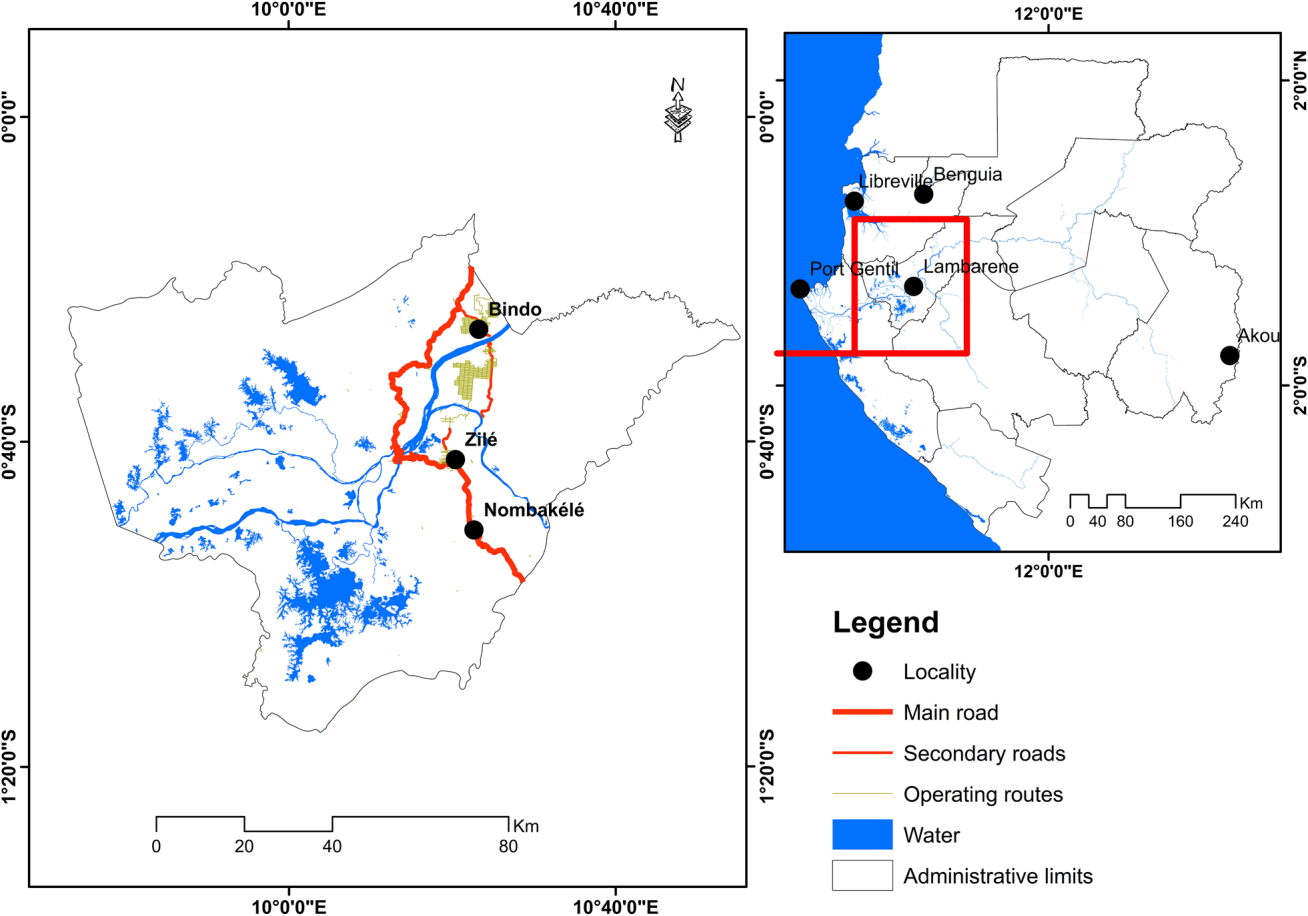
Map of the study areas

The study was carried out from May 2017 to August 2018 in three villages of Moyen Ogooué Province: Zilé (−0.703910, 10.340140), Nombakélé (−0.847490, 10.378100) and Bindo (−0.436095, 10.387816) (Fig. 1). Zilé is an area located approximately 12 km from Lambaréné and is surrounded by forest and rubber plantations. The houses, which are the homes of the plantations’ workers and families, are constructed of concrete materials. Bindo is located approximately 61 km from Lambaréné, in an area of palm tree plantations and forest. The houses are constructed of concrete materials. Nombakélé is located along the National 1 road. The inhabitants of Nombakélé have diverse occupations, and the houses are mostly built with wooden planks.

Precipitation data were obtained from the World Weather Online website [14]. During the study period, Moyen Ogooué Province was subjected to a prolonged rainy season stretching from October to May, with a small decline in precipitation from January to February, with monthly precipitation > 200 mm. The dry season extended from June to September, with monthly rainfalls of < 200 mm.

### Mosquito collection and identification

Mosquitoes were collected in both the rainy and dry seasons by overnight human landing catches (HLC), indoor and outdoor. A total of four rounds of collections were performed in the three study areas, two during the rainy seasons and two during the dry seasons, with the exception of Zilé where three collections were performed in the rainy seasons and one during the dry seasons. The mosquitoes were collected during 2 nights in each month of collection. Collectors were trained and informed by the investigators of the procedure and of the associated risks. All collectors signed an informed consent form. They were instructed to contact the team in case they developed any symptoms of malaria during the 4 weeks following the HLC. The HLC method was used in this study after the results of a pilot test aimed at assessing the efficacy of CDC-light traps in catching mosquitoes were not satisfactory.

Four collectors were appointed each night at each collection point, two indoor and two outdoor at least 5 m away from the houses. Mosquito collections were performed during 2 nights in two selected houses at each of the study locations from 1800 hours to 0600 hours. Each collection period was divided into a 50-min collection time followed by a 10-min break, and this schedule was repeated for the duration of the night. Collectors switched houses and switched from indoor to outdoor every hour to minimize any bias due to the skills of the collectors or their attractiveness to mosquitoes.

Mosquitoes that landed on the exposed legs of the collectors were collected in glass tubes and pooled per collection hour.

Mosquitoes were transferred back to the Medical Entomology Laboratory of the Centre de Recherches Médicales de Lambaréné for morphological identification. *Anopheles* mosquitoes were identified using the morphological keys of Gillies and de Meillon [15] and Gillies and Coetzee [16]. Following morphological identification, the mosquitoes were preserved at − 20 °C in silica gel in Eppendorf tubes until transferred to the Institute of Tropical Medicine in Tübingen, Germany for further processing.

The mosquitoes were dissected, and the head/thorax separated from the abdomen. The head/thorax was ground in a FastPrep-24™ 5G sample disruption instrument and lysis system (MP Biomedicals LLC, Irvine, CA, USA), and DNA was extracted using the QIAamp DNA Mini and Blood Mini Kit (Qiagen®, Hilden, Germany) and the Quick-DNA Tissue/Insect Miniprep Kit (Zymo Research Corp., Irine, CA, USA). Mosquitoes that were morphologically identified as *An. gambiae* sensu lato (s.l.) were further analysed by PCR for species identification using the PCR-SINE200 protocol of Santolamazza et al. [17]. The PCR products were then electrophoresed in a 1.5% agarose gel.

### Detection of *Plasmodium* spp. sporozoites

The extracted DNA was used to screen *Anopheles* mosquitoes for the presence of sporozoites using the protocol of Bass et al*.* [18] with slight modifications. This protocol allows for the simultaneous identification of *Plasmodium* spp. using one set of primers (PlasF: 5′-GCT TAG TTA CGA TTA ATA GGA GTA GCT TG-3′; PlasR: 5′-GAA AAT CTA AGA ATT TCA CCT CTG ACA-3′) and two probes, one labelled with the FAM fluorophore (Falci+; 5′-TCT GAA TAC GAA TGT C-3′) for the detection of *P. falciparum* and one labelled with the HEX fluorophore (OVM+; 5′-CTG AAT ACA AAT GCC-3′) for the detection of *Plasmodium ovale*, *P. vivax* and *P. malariae*. TaqMan assays were performed in the LightCycler 480 Instrument II system (Roche Applied Science, Penzburg, Germany). The cycling conditions consisted of an initial denaturation at 95 °C for 5 min, followed by 40 cycles at 95 °C for 15 s and 60 °C for 1 min.

The samples with positive assay results according to Bass et al. [18] were further differentiated using the nested qPCR protocol from Groger et al. [19] that allows for the differential identification of *Plasmodium* spp. The positive samples were pre-amplified using primers from Snounou et al. [20], and the PCR products were used as templates in a single-plex qPCR assay for each of the five species of* Plasmodium* that can cause malaria in humans (*P. falciparum*, *P. malariae*, *P. ovale curtisi*, *P. ovale wallikeri* and *P. vivax*) using previously described primers and probes [19]. The cycling conditions consisted of polymerase activation at 95 °C for 5 min, followed by 45 cycles of 95 °C for 10 s and 60 °C for 30 s.

### Identification of insecticide resistance genes

A subsample of 118 mosquitoes were selected randomly and screened for the knockdown resistance gene (*Kdr*) and *Ace-1* genes using the protocol of Bass et al. [22] with slight modifications. This protocol enables the detection of knockdown mutations and wild-type (WT) alleles in two separates assays using one set of primers (kdr-forward: 5′-CAT TTT TCT TGG CCA CTG TAG TGA T-3′; kdr-reverse: 5′-CGA TCT TGG TCC ATG TTA ATT TGC A-3′) and three probes. One of the probes was labelled with the HEX fluorophore and was used to detect the WT allele (5′-CTTACGACTAAATTTC-3′) and the remaining two probes were labelled with the FAM fluorophore for the detection of the resistant alleles Knockdown West (KdrW; 5′-ACG ACA AAA TTT C-3′) and Knockdown East (KdrE; 5′-ACG ACT GAA TTT C-3′). The cycling conditions consisted of an initial denaturation at 95 °C for 10 min, followed by 40 cycles at 95 °C for 10 s and 65 °C for 45 s.

The detection of the insensitive acetylcholinesterase (iAChe) mutation was performed using an assay that enables the WT allele and the mutant allele (S119) to be distinguished. The protocol uses one set of primers (ACE1-F: 5′-GGC CGT CAT GCT GTG GAT-3′; ACE1-R: 5′ -GCG GTG CCG GAG TAG A-3′) and two probes, one labelled with the HEX fluorophore for the detection of the susceptible allele (Ace1G119; 5′-TTC GGC GGC GGCT-3′) and one labelled with the FAM fluorophore for the detection of the resistant allele (Ace1S119; 5′-TTC GGC GGC AGC T-3′). The cycling conditions consisted of an initial denaturation at 95 °C for 10 min, followed by 40 cycles at 95 °C for 10 s and 60 °C for 35 s.

### Internal transcribed spacer 2 sequencing

Thirty-seven samples that failed to amplify using the PCR-SINE200 approach were amplified using the internal transcribed spacer 2 (ITS2) gene [22]. A subset of samples (*n* = 16) identified either by molecular or morphological methods were also sequenced. As primers, the 5.8S ATC ACT CGG CTC GTG GAT CG and 28S ATG CTT AAA TTT AGG GGG TAGTC were used. The cycling conditions consisted of 95 °C for 2 min, 30 cycles of 95 °C at 30 s, 50 °C at 30 s and 72 °C for 1 min, with a final extension of 72 °C for 5 min. The PCR products were electrophoresed in a 1.5% agarose gel to confirm that the samples were amplified. The PCR products were cleaned using ExoSAP (Thermo Fisher Scientific, Waltham, MA, USA) and sequenced using Sanger sequencing.

### Sequence analysis for species identification

The sequences were cleaned and analysed with Bioedit v. 7.2.5. The consensus sequences generated were blasted in the NCBI Genbank. Multiple sequence alignment was performed using MUSCLE in MEGA v.10.2.6 with default parameters.

The evolutionary history was inferred by using the maximum likelihood method and Kimura 2-parameter model [23]. The bootstrap consensus tree was inferred from 1000 replicates [24] and taken to represent the evolutionary history of the taxa analysed [24]. A discrete Gamma distribution was used to model evolutionary rate differences among sites (5 categories (+*G*, parameter = 63919)). Codon positions included were first, second, third positions and non-coding sites. Evolutionary analyses were conducted in MEGA [25].

### Statistical analysis

Human biting rate (HBR) was calculated by dividing the total number of *Anopheles* mosquitoes collected by the number of collectors multiplied by the number of collection nights. The EIR was calculated by multiplying the HBR by the sporozoite rate. The seasonal daily EIRs were calculated by multiplying the average HBRs and sporozoite rate of the collections carried out for each season in each study site. The seasonal monthly EIRs were determined by multiplying the seasonal daily EIRs by the number of days in each season (243 days for the rainy season [October–May] and 122 days for the dry season [June–September]). For this study, we performed two types of analysis: a descriptive analysis and a univariate explanatory analysis. The statistical analyses were carried out using R version 4.0.2 with a two-sided *P* < 0.05% indicating significance. Graphical presentation of data was done using GraphPad Prism Version 8.4.0 (GraphPad Software Inc.) A descriptive analysis was carried out on all the study data and the results were expressed as proportions. To achieve the objectives, logistic regression analysis was used to compare the number of mosquitoes collected per site and per season and the indoor/outdoor collections using binary data. We used the Pearson Chi-square (*χ*^2^) test to compare the HBRs between the three sites and Tukey’s method for multiple comparison.

## Results

### Human landing catches

A total of 1235 *Anopheles* spp. mosquitoes were collected in Bindo, Nombakélé and Zilé (Table 1). Overall, the highest HBR was recorded in Zilé, with 25 bites per person per night (b/p/n) (95% confidence interval [CI] 11.3–38.7), resulting in the collection of 800 mosquitoes, 64.8% of the total number of mosquitoes collected throughout the study period (*χ*^2^ = 22,558.4,* df* = 3, *P* < 0.0001). In Bindo and Nombakélé, the HBR was on average 11.25 (95% CI 3.6–18.9) and 2.34 (95% CI 0–5.5) b/p/n, respectively, yielding a total of 360 (29.1%) mosquitoes collected in Bindo and 75 (6.1%) mosquitoes in Nombakélé (Table 2). *Anopheles gambiae* s.l. was the most abundant species collected in the three areas, with a total of 1207 samples collected (97.7%). Other species collected were *An. moucheti* (*n* = 21, 1.7%) and *An. coustani* (*n* = 7, 0.6%).

**Table 1:** *Anopheles* species collected in Bindo, Nombakélé and Zilé

*Anopheles* spp.	Number of *Anopheles* spp. captured			
	Bindo	Nombakélé	Zilé	Total
*An. coustani*	–	1	6	7
*An. moucheti*	4	–	17	21
*An. gambiae* s.l.	356	74	777	1207
*An. gambiae* s.s	189 (97.4%)	65 (98.5%)	692 (98.7%)	946
*An. coluzzii*	4 (2.1%)	1 (1.5%)	9 (1.3%)	14
*An. gambiae/An. coluzzii*	1 (0.5%)	–	–	1

*s.l.* sensu lato,* s.s.* sensu stricto

**Table 2:** Summary of entomological indicators of malaria transmission in Bindo, Nombakélé and Zilé

Collection site	Human biting rate (b/p/n) [95% CI]	Sporozoite rate (%)	Entomological Inoculation rate			
			Night (ib/p/night)	Monthly (ib/p/month)	Seasonal (ib/p/number of months)	Annual (ib/p/year)
*Bindo*						
July 2017	0.13 [0; 0.52]	0	0	0	–	–
August 2018	0.63 [0; 1.62]	0	0	0	–	–
Dry season	0.38 [0; 0.81]	0	0	–	0	–
December 2017	30.88 [10.91; 50.84]	2.3	0.71	22.01	–	–
May 2018	13.38 [2.71; 24.04]	0	0	0	–	–
Rainy season	22.13 [11.09; 33.16]	1.5	0.33	–	80.2 ib/p/8 months	–
Annual average	–	–	–	–	–	80.2
*Nombakélé*						
July 2017	0.13 [0; 0.52]	0	0	0	–	–
July 2018	0	0	0	0	–	–
Dry season	0.06 [0; 0.20]	0	0	–	0	–
November 2017	9.25 [0; 24.41]	1.5	0.14	4.2	–	–
May 2018	0	0	0	0	–	–
Rainy season	4.63 [0; 11.28]	1.5	0.07	–	17 ib/p/8 months	–
Annual average	–	–	–	–	–	17
*Zilé*						
August 2017	4.38 [3.01; 5.73]	13.8	0.60	18.6	–	–
Dry season	4.38 [3.01; 5.73]	13.8	0.60	–	73.2 ib/p/4 months	–
May 2017	27.38 [7.75; 47.00]	1.5	0.41	12.71	–	–
November 2017	62.50 [40.08; 84.92]	2.7	1.69	50.70	–	–
February 2018	5.75 [0; 12.26]	0	0	0	–	–
Rainy season	31.88 [15.10; 48.65]	2.2	0.70	–	170.1 ib/p/8 months	–
Annual average	–	–	–	–	–	243.3

*b/p/n* Bites per person per night,* CI* confidence interval, *ib/p/night* infective bites per person per night

### Molecular identification of *Anopheles* spp.

Of the 1003 *An. gambiae* s.l. identified using molecular methods, 42 samples could not be analysed molecularly. *Anopheles gambiae* sensu stricto (s.s.) was the predominant species collected, comprising up to 97.4% (189/194), 98.5% (65/66) and 98.7% (692/701) of the mosquitoes identified in Bindo, Nombakélé and Zilé, respectively. *Anopheles coluzzii* (*n* = 14) was the second most common species identified and was found in proportions of < 2% in the three areas. One sample collected in Bindo was identified as a hybrid between *An. gambiae*/*An. coluzzii*.

### Species identification by sequencing

**Fig. 2:**
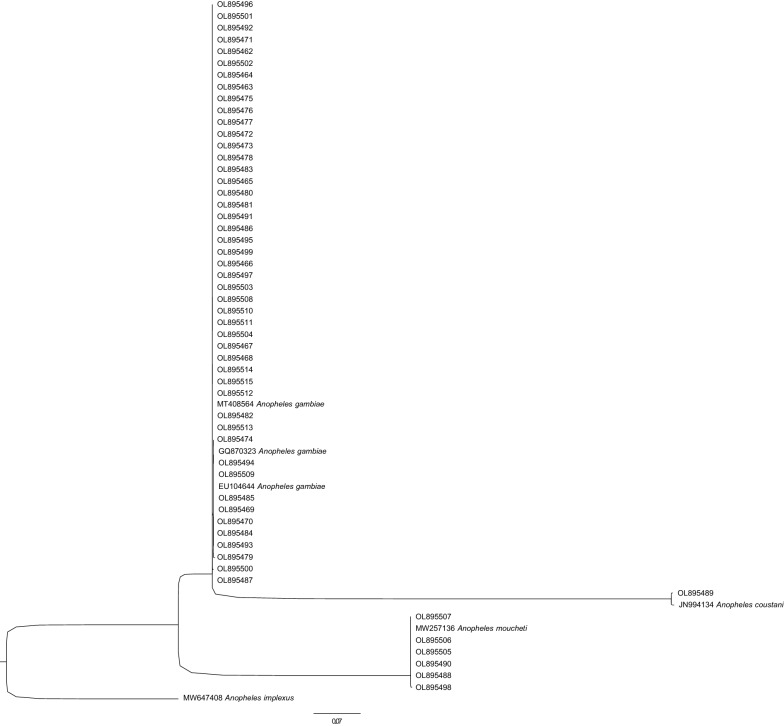
Maximum-likelihood tree of *Anopheles* ITS2 sequences. Abbreviations: ITS2, Internal transcribed spacer 2

The ITS2 sequences from 36 of the 37 samples that were morphologically identified as *An. gambiae* s.l., but which could not be identified using the SINE200 protocol, had a 99–100% identity to *An. gambiae* (Additional file 1)*.* Two samples identified as *An. gambiae* s.l. and *An. moucheti* were identified as *An. moucheti* and *An. gambiae* s.l., respectively, after ITS2 sequencing. A phylogenetic analysis was performed using the closest hits in Genbank. The sequences obtained from the samples that failed to amplify formed a clade with a high bootstrap support of 96% that included the molecularly identified samples (*An. gambiae* s.s. and *An. coluzzii*) from our collections. Two other distinct clades were formed by samples morphologically identified as *An. moucheti* and *An. coustani* (Fig. 2).

### Seasonal variations

**Fig. 3:**
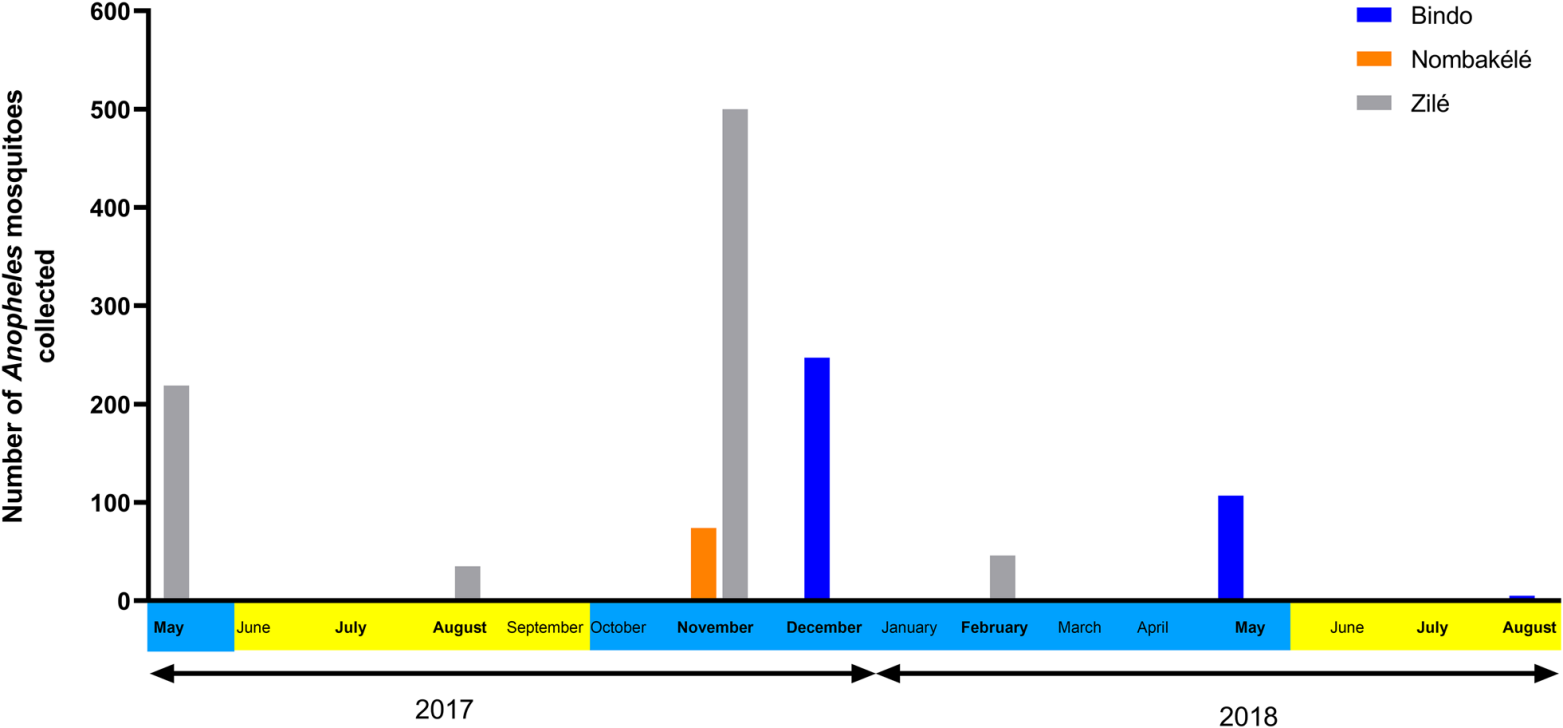
Number of *Anopheles* spp. collected according to seasons in Bindo, Nombakélé and Zilé. Months shown in bold represent the months of collections. Blue and yellow shading indicates the rainy seasons and the dry seasons, respectively

More mosquitoes (1193/1235) were collected during the rainy seasons than during the dry seasons in all three localities (generalized linear model [GLM], *P* < 0.0001) (Fig. 3). The effect of season was particularly strong at Bindo where the HBR decreased from 22.13 b/p/n (95% CI 11.09–33.16) during the rainy season to 0.38 b/p/n (95% CI 0–0.81) during the dry season (Table 2). In Nombakélé, the HBR decreased from 9.25 b/p/n (95% CI 0–24.41) in the rainy season to 0.06 b/p/n (95% CI 0–0.20) in the dry season. In Zilé, the HBR during the rainy seasons was 31.88 (95% CI 15.10–48.65) b/p/n and dropped to 4.38 (95% CI 3.01; 5.73) b/p/n during the dry season (Table 2). However, in Zilé the HBR dropped in February 2018 (5.75 b/n/p) compared to the collections carried out in November 2017 (62.5 b/n/p) and May 2017 (27.38 b/n/p).

Similar to the findings for *An. gambiae* s.l., more *An. moucheti* (*n* = 12 vs. *n* = 1) and *An. coustani* (*n* = 6 vs. *n* = 1) were collected during the rainy seasons than during the dry seasons. The dominant species during the two seasons were *An. gambiae* s.l.

### Hourly collection and biting behaviour

**Fig. 4:**
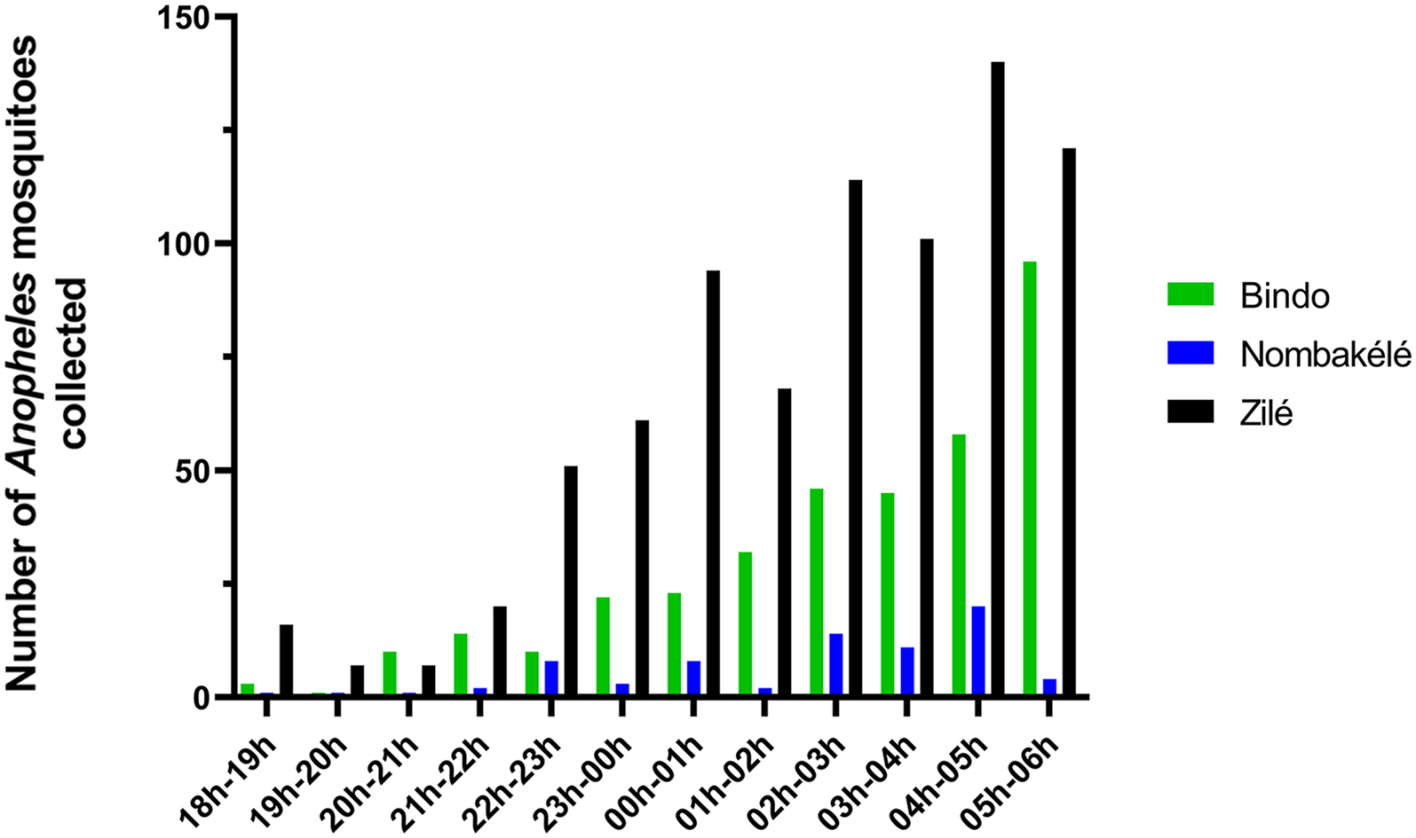
Hourly distribution of *Anopheles* mosquito bites in Bindo, Nombakélé and Zilé

In all three study areas, more mosquitoes (80.73%) were collected during the second half of the night (from midnight [00 h] to 0600 hours) than during the first half (0600 hours to midnight [00 hours]). There was a steady increase in the number of bites from *An. gambiae* s.l throughout the night up to early morning when the peak biting times were recorded. Specifically, the peak biting times in Nombakélé and Zilé were between 0400 hours and 0500 hours, and in Bindo, between 0500 hours and 0600 hours (Fig. 4). The number of *An. moucheti* and *An. coustani* was very small for a clear pattern to be observed, but similar to the biting pattern observed for *An. gambiae* s.l., most of these two species were collected during the second half of the night.

**Fig. 5:**
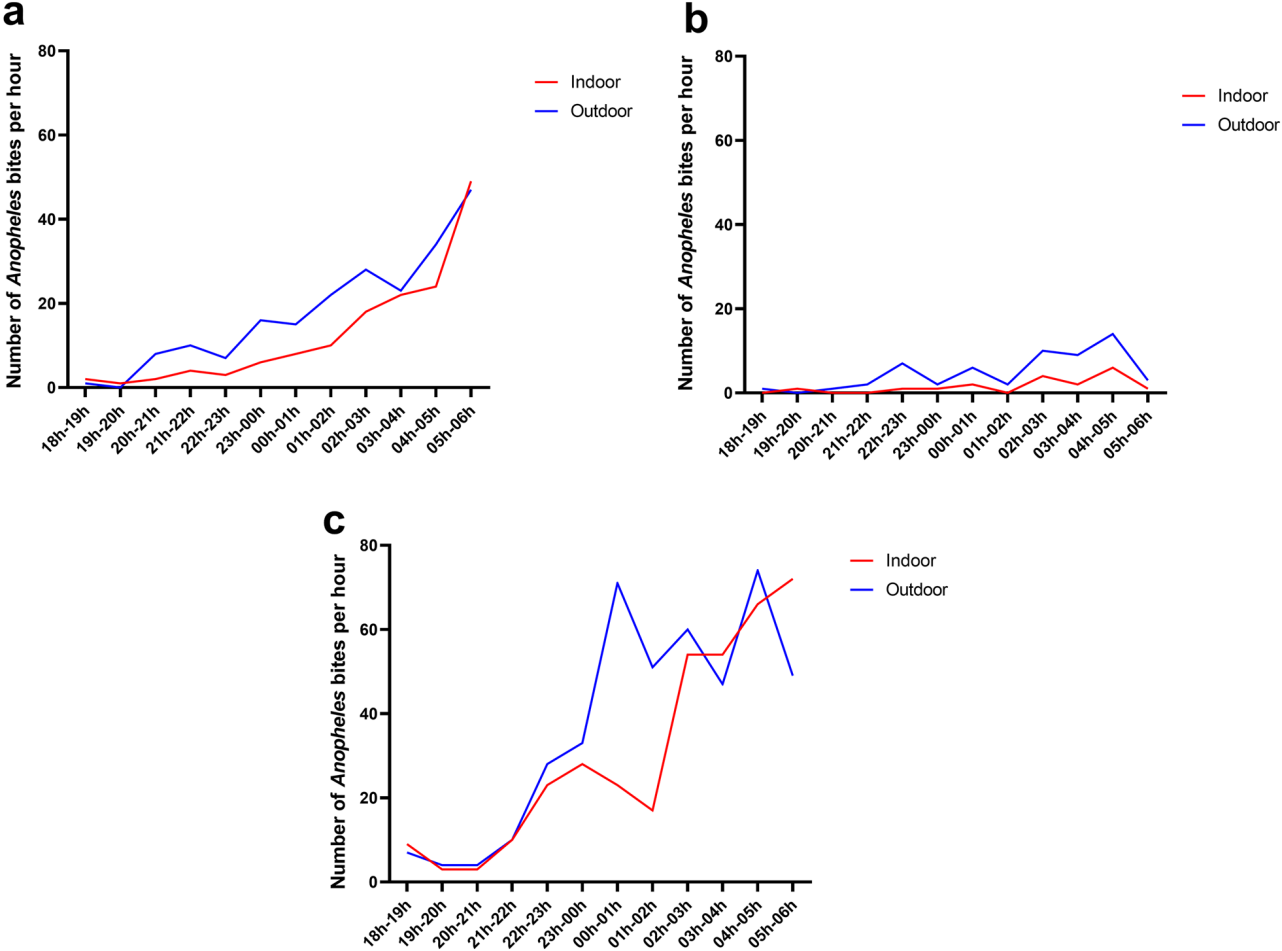
Hourly distribution of *Anopheles* mosquito bites indoor and outdoor in Bindo (**a**), Nombakélé (**b**) and Zilé (**c**)

*Anopheles* spp. collected in the three study areas exhibited a highly exophagic behaviour (Fig. 5). Consistently higher proportions of mosquitoes were collected outdoor than indoor in Bindo (58.6 vs. 41.4%; GLM, *P* ˂ 0.05), Nombakélé (76 vs. 24%; GLM, *P* ˂ 0.05) and Zilé (55 vs. 45%; GLM, *P* ˂ 0.05).

### Sporozoite rate and EIR

Out of the 1166 *Anopheles* mosquitoes screened, 26 (2.2%) were infected with *Plasmodium* spp. Of the 26 sporozoite-positive mosquitoes, 18 (69.2%) were infected with *P. falciparum*, four with *P. malariae* (15.4%), three (11.5%) with *P. ovale curtisi* and one (3.8%) with *P. ovale wallikeri*. *Plasmodium* spp. transmission was detected in three (May 2017, August 2017 and November 2017) of the four collections carried out in Zilé (Table 2). In Bindo and Nombakélé, infected mosquitoes were found only in collections performed in December 2017 and November 2017, respectively (Table 2). The highest infection rate was recorded during a collection carried out in the dry season in Zilé, with four of 29 (13.8%) *Anopheles* mosquitoes infected (Table 2).

In Bindo and Nombakélé, the annual average EIR was 80.2 and 17 infective bites/person/year (ib/p/y), respectively (Table 2). These EIRs were recorded exclusively in collections carried out between November and December and were similar to the average number of infective bites received by a person in Bindo (80.2 ib/p/8 months) and Nombakélé (17 ib/p/8 months) during the rainy season. The daily EIR in Bindo and Nombakélé was 0.71 and 0.15 infective bites per person per night (ib/p/n), respectively (Table 2).

In Zilé, the annual average EIR was estimated to be 243.3 ib/p/y. The EIR in Zilé was 2.3-fold greater during the rainy season (168 ib/p/8 months) than during the dry season (72 ib/p/4 months) (Table 2). The highest daily EIR (1.69 ib/p/n) was recorded in November 2017, yielding a monthly average of 50.70 ib/p/month. There was a drop in the daily EIR to 0.41 ib/p/n in May 2017 (rainy season) which was lower than the one recorded in August 2017 (0.60 ib/p/n) (Table 2).

### *Anopheles* resistance genes

A total of 118 *An. gambiae* s.l. were randomly selected for screening for the presence of the *Kdr* and *Ace-1*^*R*^ alleles. Of the 117 *An. gambiae* s.l. that were successfully amplified, 116 (99%) were either heterozygous or homozygous for the* Kdr* *L1014F* and *L1014S* mutations, which are associated with resistance to dichlorodiphenyltrichloroethane (DDT) and pyrethroids. Of the 116 *An. gambiae* s.l. carrying resistance mutations, 67 (57.3%) and 10 (8.5%) were homozygous for the *L1014F* and *L1014S* mutation, respectively, while 39 (33.3%) were heterozygous for both mutations. One *Anopheles* mosquito was homozygous for the WT allele *L1014L*. The *L1014F* mutation (73.9%) was the most common mutation found in mosquitoes, followed by the *L1014S* (25.2%) and *L1014L* (0.9%) mutations. Allelic frequencies between the three villages were similar.

All of the mosquitoes were homozygous for *Ace1G119*, which is the WT (susceptible) allele for resistance to carbamates and organophosphates.

## Discussion

Accurate and up-to-date data are key to the implementation of vector control measures that are efficient at reducing malaria transmission. To fill the gap in knowledge of malaria vectors in Gabon, we assessed the EIR as a measure of malaria transmission, as well as the distribution of insecticide resistance genes in *Anopheles* mosquitoes in three rural areas of Moyen Ogooué Province. Rural areas are usually hotspots of malaria transmission due to the abundance of breeding sites and the absence of adequate primary health care facilities.

ITS2 sequencing confirmed the presence of three groups of* Anopheles* species, namely *An. gambiae* s.l., *An. moucheti* and *An. coustani*, in accordance with the respective species identification based on morphological characters. *Anopheles gambiae* s.s. and *An. coluzzii* were the only species of the *An. gambiae* complex present, with the former being the dominant vector species, as previously reported in other areas of Gabon [8, 9, 11]. One sample was identified as a hybrid between *An. gambiae* s.s. and *An. coluzzii*. Hybrids of these two distinct species can occasionally be found, usually in frequencies of < 1% [26]. Other collected species, such as *An. moucheti* and *An. coustani*, may be acting as secondary vectors. The failure to amplify samples identified by sequencing of the ITS2 region as *An. gambiae* s.l. could be due to technical reasons.

Zilé had the highest HBR, followed by Bindo and Nombakélé. The high HBRs found in our study were expected based on previous collections performed in rural areas. The high HBRs is rural areas of Gabon compared to urban area are due to the availability of breeding sites for malaria vectors in the former, as reported previously [8, 9]. That Zilé was home to permanent breeding sites established through human activities could explain the high density of vectors found in this area. In addition, the lack of implementation of comprehensive vector control measures could also explain the high HBRs recorded in our study areas. Gabon has not yet implemented a mass distribution campaign of long-lasting insecticidal nets (LLINs), unlike other countries in sub-Saharan Africa [27–31]. The low LLIN ownership is a result of the targeted policy that has focused solely on the provision of ITNs to pregnant women and children aged < 5 years [1].

*Anopheles* density was significantly higher during the rainy seasons than during the dry seasons, which was expected as there is an increase in the availability of breeding sites for mosquitoes during the rainy season. This is especially true for species like *An. gambiae* s.s., the dominant species in our study areas, which has been shown to prefer temporally variable and rain-dependent breeding sites [32]. Although, the proportion of *An. coluzzii* was < 2%, the species composition of the *Anopheles gambiae* complex should be monitored regularly as a shift in species composition may have epidemiological consequences, such as year-round malaria transmission [33]. *Anopheles coluzzii* prefers long-lasting breeding sites resulting from anthropogenic activities [32]. Most *Anopheles* mosquitoes were collected during the second half of the night (> 80%), with peak biting times early in the morning. This period of the night corresponds to the time when people are sleeping, thereby presenting a reduced risk from host defensive behaviour. This late night/early morning biting behaviour is a trait well known for *An. gambiae* that may increase their ability to transmit malaria [3, 34].

*Anopheles gambiae* s.l. exhibited a highly exophagic behaviour, and all a higher number of all three *Anopheles* species was consistently collected outdoor than indoor in the three study sites, as also previously reported in Libreville [9]. This exophagic behaviour may preclude the efficacy of indoor-focused vector control interventions, such as LLINs and IRS, to significantly reduce malaria transmission [35]. The observed exophagic behaviour in the absence of large-scale vector control interventions may be due to adaptations of local *Anopheles* spp. to human sleeping behaviour in combination with physiological resistance to insecticides, which are potential exacerbators of outdoor biting [36]. A growing number of studies have reported the switch in mosquito feeding behaviour from indoor to outdoor biting following vector control interventions. This switch may have implications on the current way interventions are designed, targeting mosquitoes at the source or while resting and feeding upon humans or livestock outside of houses [5].

*Anopheles gambiae* s.s. was the sole species infected with *Plasmodium* spp. Infected mosquitoes were found in three of the four collections performed in Zilé, while in Bindo and Nombakélé they were found only in collections performed during the rainy season (October–May). *Plasmodium falciparum* was found to be the most prevalent species infecting mosquitoes although there was a substantial proportion of infections by non-falciparum species. Specifically, *P. malariae* was the second most common species infecting mosquitoes, followed by *P. ovale* curtisi and *P. ovale wallikeri*, respectively. This distribution of *Plasmodium* species is similar to that reported in humans from rural settings of Gabon [37]. In comparison, no mosquito was found to be infected with more than one species although a high prevalence of coinfections in humans has been reported from neighbouring areas [37]. This finding suggests that people living in those areas develop infections concurrently following sequential bites from mosquitoes infected with different *Plasmodium* spp. and that mosquitoes from this area have the tendency to be infected by only one parasite species at a time after feeding on coinfected individuals. Our results should draw attention to these non-falciparum species as this is the first study to screen for all *Plasmodium* species in mosquitoes in Gabon; previous studies were based on *P. falciparum* circumsporozoite protein determined by enzyme-linked immunosorbent assay [6–9, 12]. Overall, the sporozoite rate was 2.3%, with the highest sporozoite rate (13.8%) surprisingly recorded during a collection carried out during the dry season in Zilé. Similar results were reported in Thailand by Rosenberg et al. [38] and were attributed to higher vector survival rates of mosquitoes during the dry season.

The EIR is used to measure the intensity of transmission of *Plasmodium* spp. by anopheline vectors [39]. In the present study, the transmission of malaria was different across study sites, with almost a perennial transmission in Zilé and intermittent transmission in Bindo and Nombakélé. The major contribution of the period between October to December to the overall burden of malaria transmission in our study areas was exemplified by the fact that the highest EIR in Zilé as well as the sole EIRs in Bindo and Nombakélé were recorded in collections carried out during this period. The annual average EIR (243.3 ib/p/y) recorded in Zilé was one of the highest ever recorded in Gabon and should be associated to a high infection rate in populations living in this area. Indeed, Beier et al. [41] reported that annual EIRs of ≥ 200 are regularly associated with a > 80% prevalence of *P. falciparum* in humans. Although the average annual EIRs recorded in Bindo and Nombakélé were lower than that in Zilé, the former suggest a *P. falciparum* prevalence in humans of at least 50% [40]. Aside from the availability of breeding sites as mentioned above, this high transmission intensity in Zilé may be a consequence of the high prevalence of helminth infections, such as *Schistosoma haematobium* and *Trichuris trichiura* or hookworm, in populations living in these area compared to populations living in Bindo and Nombakélé [41]. These helminths, especially *S. haematobium*, have been shown to have an effect on *P. falciparum* infections in humans by increasing *P. falciparum* incidence, thus increasing its transmission intensity either alone [42–44] or in synergy with other helminths, such as *Trichuris trichiura* or hookworm [41].

Previous reports have shown the presence of the *Kdr* mutations in local vector populations [8, 9, 17, 45]. Libreville was the first coastal West African location where the presence of both *L1014F* (*Kdr-w*) and *L1014S* (*Kdr-e*) was observed [45]. These mutations were subsequently also found in mosquito populations from other areas, such as Benguia [46], Port-Gentil and Libreville [8] and Mouila [10]. The genotypic and allelic frequencies of the *Kdr* mutations observed in *An. gambiae* collected in the present study are similar to frequencies reported in previous studies in Gabon and suggest the presence of high level of resistance in these mosquito populations to pyrethroids and DDT [8, 9, 17, 45]. This potential resistance to pyrethroids, in the absence of a mass distribution of LLINs, may be driven by the use of these insecticides in agriculture, as previously described [47, 48]. However, our finding that all of the mosquitoes screened carried the susceptible allele for carbamate and organophosphate may hint to the susceptibility of mosquitoes to these classes of insecticides, suggesting that a combination strategies may be used as a tool to circumvent the effect that pyrethroid resistance may have on the efficacy of LLINs. Such studies have been conducted in Burkina-Faso [49] and Tanzania [50], with the results showing that simultaneous use of LLINs and net wall hangings treated with organophosphate improved malaria control.

Our study has a number of limitations, which mainly include the small sample size in terms of mosquitoes collected as well as the number of collections which did not allow us to fully assess the effect of seasonal variations on mosquito populations. In addition, the use of qPCR to screen for mosquito infections did not allow us to determine with confidence the infective status of mosquitoes. Although Foley et al. [51] reported that bisection of mosquitoes anterior to the junction of the thorax and abdomen eliminates the risk of false positives, it has been demonstrated that this risk is not totally eliminated, especially when using very sensitive PCR protocols [52].

## Conclusion

The assessment of *Plasmodium* spp. distribution based on the results of the present study revealed a high prevalence of non-falciparum species in the mosquitoes collected, which should draw more attention to their contribution to the malaria burden in Gabon, particularly in Moyen Ogooué Province. From our results, it is obvious that the transmission of malaria was heterogenous in the three areas, where Zilé could be considered to be an area of high transmission. The combination of exophagic behaviour of mosquitoes and the high frequencies of *Kdr* mutations before the implementation of a mass distribution of LLINs may significantly impede the success of such a strategy to durably curb malaria transmission. Thus, there is a need to adopt vector control strategies that will include the use of other insecticide classes and new vector control tools.

## Supplementary Information

**Additional file 1.** Sequence identification of *Anopheles* sp. using ITS2.

## Data Availability

Raw data are archived and available on request from the corresponding author. The ITS2 sequences found in this study were deposited in the GenBank database with accession numbers OL895462–OL895515.

## Abbreviations

DDT: Dichlorodiphenyltrichloroethane
EIR: Entomological inoculation rate
HBR: Human biting rate
HLC: Human landing catches
*iAChE*: Insensitive acetylcholinesterase
IRS: Indoor residual spraying
ITN: Insecticide-treated net
ITS2: Internal transcribed spacer 2
*Kdr*: Knockdown resistance gene
LLIN: Long-lasting insecticidal net
